# Improving emergency department care for adults presenting with mental illness: a systematic review of strategies and their impact on outcomes, experience, and performance

**DOI:** 10.3389/fpsyt.2024.1368129

**Published:** 2024-02-29

**Authors:** Elizabeth E. Austin, Colleen Cheek, Lieke Richardson, Luke Testa, Amanda Dominello, Janet C. Long, Ann Carrigan, Louise A. Ellis, Alicia Norman, Margaret Murphy, Kylie Smith, Donna Gillies, Robyn Clay-Williams

**Affiliations:** ^1^ The Centre for Healthcare Resilience and Implementation Science, Australian Institute of Health Innovation, Faculty of Medicine, Health and Human Science, Macquarie University, Macquarie, NSW, Australia; ^2^ Centre for the Health Economy, Macquarie University Business School, Macquarie University, Macquarie, NSW, Australia; ^3^ Western Sydney Local Health District, New South Wales Health, Sydney, NSW, Australia; ^4^ Emergency Care Institute, New South Wales Agency for Clinical Innovation, New South Wales Health, Sydney, NSW, Australia; ^5^ Quality and Safeguards Commission, National Disability Insurance Scheme, Sydney, NSW, Australia

**Keywords:** healthcare quality, quality improvement, mental health, process re-design, equity, equality, acute care, emergency department

## Abstract

**Background:**

Care delivery for the increasing number of people presenting at hospital emergency departments (EDs) with mental illness is a challenging issue. This review aimed to synthesise the research evidence associated with strategies used to improve ED care delivery outcomes, experience, and performance for adults presenting with mental illness.

**Method:**

We systematically reviewed the evidence regarding the effects of ED-based interventions for mental illness on patient outcomes, patient experience, and system performance, using a comprehensive search strategy designed to identify published empirical studies. Systematic searches in Scopus, Ovid Embase, CINAHL, and Medline were conducted in September 2023 (from inception; review protocol was prospectively registered in Prospero CRD42023466062). Eligibility criteria were as follows: (1) primary research study, published in English; and (2) (a) reported an implemented model of care or system change within the hospital ED context, (b) focused on adult mental illness presentations, and (c) evaluated system performance, patient outcomes, patient experience, or staff experience. Pairs of reviewers independently assessed study titles, abstracts, and full texts according to pre-established inclusion criteria with discrepancies resolved by a third reviewer. Independent reviewers extracted data from the included papers using Covidence (2023), and the quality of included studies was assessed using the Joanna Briggs Institute suite of critical appraisal tools.

**Results:**

A narrative synthesis was performed on the included 46 studies, comprising pre-post (*n* = 23), quasi-experimental (*n* = 6), descriptive (*n* = 6), randomised controlled trial (RCT; *n* = 3), cohort (*n* = 2), cross-sectional (*n* = 2), qualitative (*n* = 2), realist evaluation (*n* = 1), and time series analysis studies (*n* = 1). Eleven articles focused on presentations related to substance use disorder presentation, 9 focused on suicide and deliberate self-harm presentations, and 26 reported mental illness presentations in general. Strategies reported include models of care (e.g., ED-initiated Medications for Opioid Use Disorder, ED-initiated social support, and deliberate self-harm), decision support tools, discharge and transfer refinements, case management, adjustments to liaison psychiatry services, telepsychiatry, changes to roles and rostering, environmental changes (e.g., specialised units within the ED), education, creation of multidisciplinary teams, and care standardisations. System performance measures were reported in 33 studies (72%), with fewer studies reporting measures of patient outcomes (*n* = 19, 41%), patient experience (*n* = 10, 22%), or staff experience (*n* = 14, 30%). Few interventions reported outcomes across all four domains. Heterogeneity in study samples, strategies, and evaluated outcomes makes adopting existing strategies challenging.

**Conclusion:**

Care for mental illness is complex, particularly in the emergency setting. Strategies to provide care must align ED system goals with patient goals and staff experience.

## 1 Introduction

Emergency departments (EDs) are tasked with providing high-quality, safe, and timely acute care. To meet the changing needs of the community, care safety and quality are continually assessed to identify targeted areas for improvement. Inadequately resourced mental healthcare elsewhere in the system has contributed to rising presentations to ED (1, 2), but EDs experiencing overcrowding and access block (delay in transferring the person to an admitted hospital ward bed) may be unsuitable for the management of mental illness (3). The high-stimulus environment and lengthy wait times can result in poor patient outcomes, including leaving before completion of care, or escalating patient agitation and use of restrictive interventions, including traumatic use of restraint (4, 5). Patients seeking ED care for mental illness report poor staff attitudes and knowledge, and feeling powerless to access needed care (6). Negative experiences can discourage a person from accessing care in the future (6–8). Similarly, ED staff report being inadequately prepared or resourced to provide care for some presenting mental illness, and an inability to obtain timely patient assessment by specialist mental health staff (9–11). Patient and staff experiences are reflected in ED system performance, with measures such as wait times, total ED length of stay (LoS), and left at own risk (left the ED prior to completion of care) (12) reflecting problems in care provision for adults with mental illness in the ED. Thus, an imperative exists to improve care delivery for adults presenting to ED with mental illness.

The ED has a long history of innovating practices and processes, such as clinical pathways to expedite standardised intervention, as well as expanding the ED team composition to include advanced nursing and allied health roles to improve care delivery (13). The desire to improve care for adults presenting to ED with mental illness has prompted better understanding of the characteristics of ED presentations such as deliberate self-harm (14–16), suicidal ideation (17, 18), anxiety and depression (19, 20), substance use (21), specific vulnerabilities among the homeless (22, 23) or incarcerated adults (24, 25), and symptomatology including agitation resulting in restraint (26, 27). Similarly, systematic reviews shed light on the strategies employed to improve care for adults presenting to ED with acute mental illness, exploring the effectiveness of case management (28), various liaison psychiatry models (29), as well as strategies specifically targeting frequent users (30), deliberate self-harm (31), and opioid use disorder (32). These systematic reviews provide insights into the impact of strategies to improve care delivery in the ED for individual models of care and specific patient presentations, they do not provide a comprehensive synthesis of reported strategies and their impact on system performance, patient outcomes, patient experience, and staff experience.

A 2019 scoping review by Johnston and colleagues (33) identified a wide range of strategies implemented or delivered in the ED for adults presenting with mental illness. Strategies were patient-focused, including information, education, psychotherapy, and pharmacology; staff-focused, such as education and assessment processes; or system-focused, for example, new referral processes and the capacity to case manage, with some studies focusing on staff and patients (33). While comprehensive, the 2019 review did not identify strategies to improve patient experience. Understanding and improving patient experience is essential for patient engagement and clinical outcomes (34). Consequently, identifying strategies to improve patient experience is critical for informing changes in ED care delivery. Given the increasing volume of mental health presentations (35, 36), as well as workforce constraints and increasing service demand (35), it is necessary to revisit the question of what strategies have been used to improve care delivery outcomes and experiences for adults with mental illness in the ED, to guide service adaptation.

Understanding the strategies that have been successful in improving care delivery or experience will enable ED clinicians, managers, and hospital executives to make more informed decisions about what can be done to improve care in their local context. We sought to identify interventions implemented in the ED for people presenting with mental illness as a foundation for a comprehensive programme to codesign new or adapted models of ED care for this cohort (37). Therefore, the purpose of this systematic review was to examine the research evidence provided in the peer-reviewed literature to identify the relationship between the strategies used to improve ED care delivery for adult mental illness presentations and measures of (1) system performance, (2) patient outcomes, (3) patient experience, and (4) staff experience.

## 2 Methods

The study protocol was registered in September 2023 in Prospero (CRD42023466062). The study protocol guided the review in accordance with the Preferred Reporting Items for Systematic Reviews and Meta-Analyses (PRISMA) statement (38).

### 2.1 Search strategy

A comprehensive search strategy using medical subject headings and text words for the general concepts of ED, improvement, outcomes, and mental illness was developed in consultation with a research librarian. Scopus, Ovid Embase, CINAHL, and Medline were searched on 22 September 2023 for peer-reviewed English language articles. No date limits were set. The full search strategy is shown in **Supplementary File A**.

### 2.2 Eligibility criteria

Empirical peer-reviewed research articles were included in the systematic review if they met the following criteria:

*Population*: (1) Mental health presentations [e.g., undifferentiated, suicidal, deliberate self-harm, scheduled, substance-related and addictive disorders (e.g., drug and alcohol), depressive disorders, anxiety disorders]; (2) adults; and (3) in the ED.

*Intervention*: Implemented models of care or system changes (e.g., redesigning the environment to reduce stimulation, new care pathway).

*Comparison*: Usual care or other form of care.

*Outcome*: Measures of (1) system performance (e.g., waiting time, LoS, time to treatment/assessment etc., admissions, and referrals); (2) patient outcomes (e.g., readmission, adverse events, medical errors, missing diagnosis, pain, and quality of life); (3) patient experience (e.g., patient experience, complaints, did not wait, left without being seen, and left at own risk); or (4) staff experience (e.g., staff experience, job satisfaction, and intention to stay).

Articles were excluded if they (1) reported on interventions that were conducted primarily in the pre-hospital, post-hospital, or a ward/clinic setting other than the ED; (2) involved persons under 18 years of age; (3) focused on disability or neurodiversity (e.g., autism); (4) did not report an intervention (e.g., reported only trends or characterisations), screened presentations with no accompanying intervention within the ED; (5) were literature reviews, conference poster or abstract, grey literature, and case report; or (6) were published in a language other than English.

### 2.3 Screening and data extraction

The search results were entered into EndNote citation management software (version 20.6; Thompson Reuters, New York, NY) and duplicates were removed. References were uploaded into Covidence, a subscription web-based tool for conducting screening, data extraction, and critical appraisal for systematic reviews. During the title and abstract, and full-text screening phases of the review, each article was screened independently by pairs of reviewers for inclusion according to the predefined criteria. Disagreements were resolved by an independent third reviewer.

Data were extracted by independent reviewers in the Covidence platform (2023) (39) using a customised extraction tool specifically developed for the review. The data extraction form was piloted for usability on four articles by four independent reviewers before data extraction commenced. The data extraction form included information on the country where the study was conducted, the aim of the study, the study design, the number of EDs included, the aim of the intervention, description of the intervention, number of participants, the mental illness focus, participant inclusion and exclusion criteria, participant characteristics, evaluated outcomes (i.e., system performance, patient outcomes, patient experience, and staff experience), and study limitations. The data extraction form is shown in **Supplementary File B**.

### 2.4 Risk of bias

The methodological quality of the included articles was assessed using the Joanna Briggs Institute critical appraisal tool for the study type: Checklist for Cohort Studies, Checklist for Case–Control Studies, Checklist for Analytical Cross-Sectional Studies, Checklist for Randomised Controlled Trials, Checklist for Quasi-Experimental Studies, and the Checklist for Qualitative Research Studies (40, 41). Four articles were used to pilot the critical appraisal tool. Each article was critically appraised by pairs of independent reviewers in Covidence, with disagreements resolved via discussion. Covidence does not have a mechanism for allocating a subset of studies, nor is there a mechanism to calculate a metric of inter-rater reliability for critical appraisal. Instead, reviewers are allocated a number of papers to appraise to ensure that the work is divided in a just way.

### 2.5 Data processing and analysis

Because of the heterogeneity of included articles, a narrative synthesis was conducted for this review. Data were synthesised according to the mental illness focus of the presentation and included numerical statistical summaries, textual commentaries, and tabular and graphical representations.

### 2.6 Patient and public involvement

Patients and the public were not involved in the design and conduct of this review.

## 3 Results

### 3.1 Literature search

**Figure 1:**
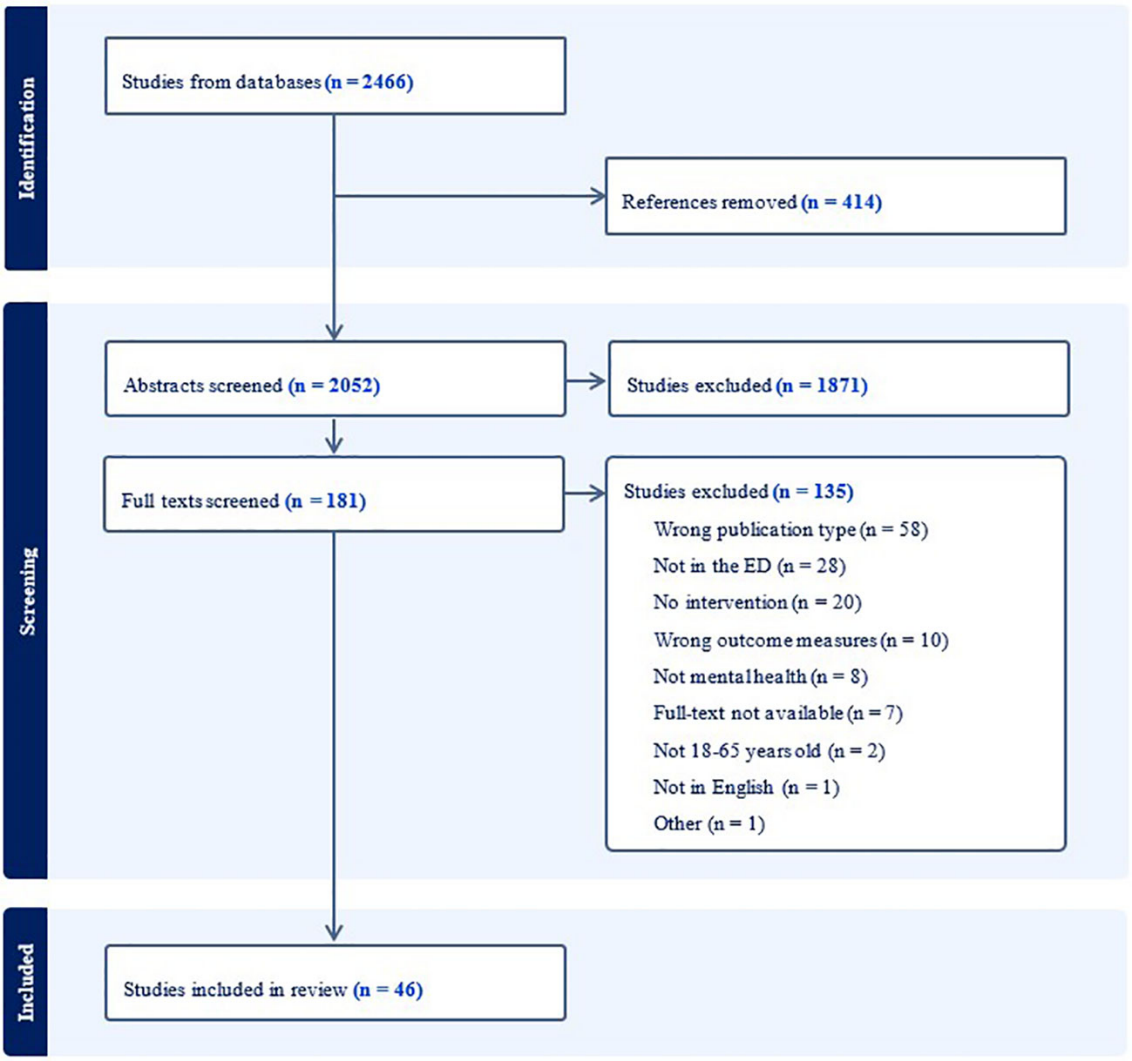
PRISMA flow diagram for study selection.

The combined searches yielded 2,466 articles, including 414 duplicate articles. Of these, 2,052 abstracts and 181 full texts were screened with 46 articles meeting the inclusion criteria. **Figure 1** depicts the PRISMA diagram for the identification, screening, and inclusion processes.

### 3.2 Description of included studies

Characteristics of the included studies are in **Supplemetary File C**. Included articles were published between 2000 and 2023 and primarily conducted in high-income countries including the USA (54%, *n* = 25), Australia (15%, *n* = 7), the UK (13%, *n* = 6), and Canada (9%, *n* = 4). One study each was conducted in Ireland, Israel, Japan, and Norway.

### 3.3 Quality assessment

Most studies were assessed as having potential flaws or limitations in their design, conduct, or analysis that could distort the results. While risk of bias is important to report, all studies were retained in this review for the potential learning that may be derived about the interventions and measures that might be tested more rigorously in subsequent studies should they have potential for the readers’ context. The outcomes from the quality assessment are shown in **Table 1**.

**Table 1:** Results of the critical appraisal.

Randomised controlled trials													
**Reference**	**Q1**	**Q2**	**Q3**	**Q4**	**Q5**	**Q6**	**Q7**	**Q8**	**Q9**	**Q10**	**Q11**	**Q12**	**Q13**
Bryan, 2018, USA (53)	Y	Y	Y	Y	N	Y	Y	Y	Y	Y	Y	Y	Y
Clarke, 2002, UK (54)	Y	Y	Y	N	N	Y	U	Y	Y	Y	Y	Y	Y
Inui-Yukawa, 2021, Japan (55)	Y	U	Y	Y	N	Y	U	Y	Y	Y	Y	Y	Y
Cohort studies													
**Reference**	**Q1**	**Q2**	**Q3**	**Q4**	**Q5**	**Q6**	**Q7**	**Q8**	**Q9**	**Q10**	**Q11**		
Maeng, 2020, USA (73)	Y	Y	Y	Y	Y	Y	Y	Y	NA	NA	Y		
Solomon, 2023, USA (52)	Y	Y	Y	N	Y	U	Y	Y	Y	U	Y		
Qualitative research													
**Reference**	**Q1**	**Q2**	**Q3**	**Q4**	**Q5**	**Q6**	**Q7**	**Q8**	**Q9**	**Q10**			
Poremski, 2016, Cananda (77)	Y	Y	Y	Y	Y	Y	Y	Y	Y	Y			
Vakkalanka, 2022, USA (82)	U	Y	Y	Y	Y	U	U	Y	U	Y			
Xanthopoulou, 2022, UK (61)	U	U	U	U	U	N	Y	Y	Y	Y			
Quasi-experimental studies													
**Reference**	**Q1**	**Q2**	**Q3**	**Q4**	**Q5**	**Q6**	**Q7**	**Q8**	**Q9**				
Adams, 2012, USA (62)	Y	U	Y	N	N	U	Y	Y	U				
Alexander, 2020 (63),	Y	NA	NA	N	Y	NA	Y	Y	Y				
Bistre, 2022, Israel (64)	N	U	N	N	NA	NA	Y	Y	Y				
Brainch, 2018, USA (65)	Y	U	Y	Y	Y	Y	Y	Y	Y				
Braitberg, 2018, Australia (66)	Y	N	Y	Y	N	Y	Y	Y	Y				
Broadbent, 2022, Australia (67)	Y	U	U	Y	N	Y	Y	U	U				
Butler, 2022, USA (43)	Y	Y	Y	Y	Y	NA	Y	Y	Y				
Clarke, 2006, Canada (68)	Y	U	Y	N	Y	NA	Y	Y	Y				
Eppling, 2008, USA (69)	Y	U	U	N	Y	NA	U	U	U				
Faude, 2023, USA (44)	N	Y	N	N	N	U	N	Y	Y				
Gertner, 2021, USA (45)	Y	U	U	Y	N	U	U	U	Y				
Johnsen, 2007, Norway (71)	Y	N	U	N	Y	N	Y	Y	Y				
Kahler, 2017, USA (46)	Y	Y	Y	N	N	NA	Y	Y	Y				
Kim, 2022, USA (56)	Y	Y	Y	N	Y	NA	Y	Y	Y				
Lauer, 2008, USA (72)	Y	U	U	N	U	Y	U	U	N				
Lepping, 2006, UK (58)	Y	U	U	N	Y	U	Y	U	U				
Lowenstein, 2023, USA (47)	Y	Y	Y	Y	Y	NA	Y	Y	Y				
Lukacs, 2023, USA (48)	Y	Y	U	N	Y	Y	Y	Y	Y				
McCurdy, 2015, USA (74)	Y	Y	Y	Y	Y	Y	Y	Y	U				
Mitchell, 2020, Australia (75)	Y	U	U	N	Y	U	Y	Y	N				
Morgan, 2000, UK (59)	Y	Y	Y	N	Y	Y	Y	Y	Y				
Murphy, 2023, USA (51)	Y	Y	Y	N	Y	N	Y	Y	Y				
Okafor, 2016, USA (76)	Y	U	Y	Y	Y	U	Y	Y	Y				
Opmeer, 2017, UK (60)	Y	Y	Y	N	Y	Y	Y	Y	Y				
Reinfeld, 2023, USA (78)	Y	NA	NA	N	Y	U	Y	Y	Y				
Simpson, 2018, USA (79)	Y	Y	Y	Y	N	Y	Y	Y	Y				
Sinclair, 2006, UK (80)	Y	Y	Y	Y	Y	NA	Y	Y	Y				
Stover, 2015, USA (81)	Y	U	Y	Y	Y	Y	Y	Y	Y				
Woo, 2007, USA (85)	Y	Y	Y	Y	Y	Y	Y	Y	Y				
Zwank, 2020, USA (87)	Y	Y	Y	N	N	NA	Y	Y	Y				
Cross-sectional studies													
**Reference**	**Q1**	**Q2**	**Q3**	**Q4**	**Q5**	**Q6**	**Q7**	**Q8**					
Gabet, 2020, Canada (70)	Y	Y	Y	Y	Y	NA	Y	Y					
Wand, 2001, Australia (83)	N	Y	Y	Y	N	NA	N	NA					

N, no; NA, not available; U, unclear; Y, yes.

### 3.4 Mental health presentations

Eleven of the 46 included articles (23.9%) focused on substance-related and addictive disorders (e.g., substance use disorder) and 9 (19.6%) focused on suicidal or deliberate self-harm, whereas 26 (56.5%) sought to address care delivery for all mental illness presentations.

### 3.5 Innovations for substance-related and addictive disorder presentations

Eleven of the 46 articles reported on strategies focusing on substance-related and addictive disorder presentations (**Table 2**). Four types of strategies were evaluated, including the ED-initiated Medications for Opioid Use Disorder (MOUD) model of care (42–45, 49–52), decision support tools (42, 46, 47), ED-initiated social support model of care (48), and discharge and transfer of care (42, 46). Overall, the ED-initiated MOUD model of care reported effects across all four domains of interest; however, not one study reported on all four. System performance effects (e.g., identification of eligible patients) were reported for three of the four strategies; all four strategies evaluated patient outcomes (e.g., engagement with formal treatment); one strategy evaluated patient experience (e.g., satisfaction); and one strategy evaluated staff experience (e.g., readiness). Eight of the 11 articles included at least one academic medical centre (42–47, 49, 51), with three of the eight articles including at least one community hospital (42, 47, 49). One of the eight articles also included a private hospital (42). Three of the 11 studies did not report the type of participating hospital (48, 50, 52). Eight of the 11 articles were conducted in urban settings (43, 44, 46–51), with one reporting an additional rural site (49). Three of the 11 studies did not report the setting as either urban or rural (42, 45, 52). Three articles reported that the participating EDs were connected with psychiatric crisis centres or psychiatric EDs (44, 45, 47).

**Table 2:** Substance-related and addictive disorder focused interventions, intervention characteristics, and their reported effects.

Intervention	Intervention characteristics	Reported EFFECTS
**ED-initiated MOUD model of care**	Opioid use disorder clinical pathways (52) designed to initiate buprenorphine/naloxone in ED, including screening, prescription, take home doses, and follow-up clinic referral (42–45, 49–51). Can include peer support (44, 45) or case management (51)	*System Performance*: no difference in median ED LoS (50), not all eligible patients received the treatment (42, 44, 45) *Patient Outcomes*: increased the number of patients engaged in formal treatment following ED visit (42–45, 49, 50, 52), improved quality of life (51) *Patient Experience*: patients were satisfied (49) *Staff Experience*: increased readiness to provide treatment (44, 49)
**Decision support tools**	Modifications to EMR to include prompts and notifications related to universal screening at triage (42, 47), patient arrival in the ED (46), measure withdrawal and guide next steps (47) such as referral, management plan, and instructions (46)	*System Performance*: increased the number of eligible patients identified (42, 47) and treatments delivered (42, 47), decreased ED visits, prescriptions, and pathology tests (46) *Patient Outcomes*: decreased hospitalisations and the number of hospital days per year (46)
**ED-initiated social support model of care**	Following substance use screening, a peer recovery coach, based in the ED, provides assistance with health system navigation, social support, resources, and SUD referral (48)	*Patient Outcomes*: decreased ED utilisation, increased engagement with resources, and increased reported abstinence at 90 days (48)
**Discharge and transfer of care**	Collaboration and communication with community providers (42) and referrals to clinics (46)*	*System Performance*: decreased ED visits, prescriptions, and pathology tests (46), increased the number of eligible patients identified and treatments delivered (42) *Patient Outcomes*: decreased hospitalisations and the number of hospital days per year (46)

ED, emergency department; EMR, electronic medical record; LoS, length of stay; *Kahler et al.’s (46) strategy included a cessation of opioid prescription from the ED.

### 3.6 Innovations for suicide and deliberate self-harm presentations

Nine of the 46 articles reported on strategies focusing on suicide and deliberate self-harm presentations. The five types of strategies comprised case management, liaison psychiatry services, deliberate self-harm models of care, and specialised units, and focused on assessment (53–56, 58–61), brief interventions (56, 60, 61), monitoring (57), care plan development (53–55), and referral to community-based support (53, 54, 56). System performance effects (e.g., wait time for assessment) were reported for three of the five strategies, four strategies reported patient outcomes (e.g., rate of self-harm), one strategy reported patient experience, and one strategy reported staff experience (e.g., favourability). A description of the characteristics of each type of suicide or self-harm strategy and their reported effect is provided in **Table 3**. Two of the nine articles included an academic medical centre and one general hospital (30, 57, 59). Six of the nine studies did not report the type of participating hospital (53–55, 58, 60, 61). Two of the nine articles were conducted in urban settings (57, 58), with one reporting a semi-rural site (59). Six of the nine studies did not report the setting as either urban or rural (53–56, 60, 61). Four articles reported that the participating EDs were connected with psychiatric services such as liaison psychiatry (56, 58–60), one reported not having access to psychiatric services (57), and four did not report on access to psychiatric services (53–55, 61).

**Table 3:** Suicide or deliberate self-harm interventions, intervention characteristics, and reported effects.

Intervention	Intervention characteristics	Reported effects
**Case management**	Case management consists of psychosocial assessment and continuous negotiated care plan provided by a mental health professional (54) or case manager (55)	*Patient Outcomes*: decrease in suicide attempts (55), decrease in self-harm (55), no reduction in readmission rate (54)
**Self-harm model of care**	Self-harm models of care include risk assessment, medical examination, specialist psychosocial assessment, referral to community mental health team (58) provided by a dedicated team (59) and can include the development of personalised response plan that includes personal warning signs, self-management, social support, reasons for living (53)	*System Performance*: increases requested and completed psychosocial assessments (58, 59) and referral rate (59) *Patient Outcomes*: reduced ED utilisation (59)
**Suicide risk monitoring protocol**	Suicide risk monitoring protocol for virtual monitoring with patients who are identified by their clinicians not to be impulsive (57)	*Patient Outcomes*: No adverse events, two no-harm incidents (57) *Staff Experience*: high favourability for virtual monitoring with a preference for virtual, but no difference between virtual and face to face monitoring in favourability (57)
**Liaison Psychiatry Service**	Liaison Psychiatry Service is a specialist service that includes psychosocial assessment (61) and brief interventions. Typically operating between Monday and Friday, 09:00–17:00 to 8:00, but have been extended to 22:00, 7 days a week (60)	*System Performance*: increase in the proportion receiving a psychosocial assessment, reduced wait time for psychosocial assessment, decreased wait time between medical and psychosocial assessment, decreased cost per attendance and cost per patient (60) *Patient Outcomes:* decreased LWBS (60) *Patient Experience*: therapeutic conversations reduce distress and instill hope, formulaic assessments that focus on risks were tedious and generic (61)
**Specialised unit within the ED**	Dedicated space with recliners (open unit concept) where each patient receives nursing evaluation, psychosocial evaluation from social workers, and psychiatric evaluation from psychiatrist. Treatment and referrals are provided (56)	*System Performance:* reduced hospital admissions, decreased ED LoS, no difference in restraint use (56) *Patient Outcomes:* reduced ED utilisation (56)

ED, emergency department; LWBS, left without being seen.

### 3.7 Innovations for mental health presentations

Twenty-six of the 46 articles reported on strategies that encompassed all mental illness presentations to the ED (**Table 4**). The 11 types of strategies implemented in ED included decision support tools [e.g., modifications to electronic medical record (EMR)] (62, 63, 67, 79), discharge and transfer of care (e.g., collaboration and communication processes) (62, 63, 81), liaison psychiatry services (e.g., included ED staff in meetings) (63), telepsychiatry (e.g., assessment) (64, 82), changes to roles and rostering (e.g., mental health professions in ED) (63, 65, 69, 73, 78, 80, 83), specialised units within ED (e.g., behavioural assessment unit) (66, 72, 75, 76, 85, 86), education and training (e.g., related to mental illness) (68), intervention teams (e.g., multidisciplinary team) (70, 84), standardising protocols (e.g., procedures for agitated behaviour management) (71, 78, 79, 86, 87), environmental design (e.g., glass doors) (74), and case management (e.g., facilitating connections with services) (77, 86). System performance effects (e.g., ED LoS and use of restraint) were reported for all 11 strategies, 7 strategies reported patient outcomes (e.g., ED utilisation), 6 strategies reported patient experience (e.g., preferences), and 8 strategies reported staff experience (e.g., confidence). Fourteen of the 26 articles included an academic medical centre (63, 65, 66, 68, 70, 74, 76, 77, 79, 83–87), and 3 of the 26 included a community hospital (62, 69, 73). Nine of the 26 studies did not report the type of participating hospital (64, 67, 71, 72, 75, 78, 80–82). Eighteen of the 26 articles were conducted in urban settings (63, 65, 66, 68–70, 72, 75–77, 79–81, 83–87), with one reporting an additional rural site (84), and two reported only rural sites (73, 82). Five of the 26 studies did not report the setting as either urban or rural (62, 64, 67, 71, 74, 78). Thirteen articles reported that the participating EDs were connected with psychiatric crisis services (62, 69–71, 74–76, 78, 79, 82, 85–87), and 13 did not (20, 63–68, 72, 73, 77, 80, 81, 83).

**Table 4:** Mental health presentation interventions, intervention characteristics, and reported effects.

Intervention	Intervention characteristics	Reported effect
**Decision support tools**	Modifications to EMR systems to include notification of discharge within the last 30 days (62), prompts to attest to completion of tasks (79), add assessment questions to identify previously used or failed care plan strategies (62), psychiatric medication chart to include “prescribe as required” and provide advice (63), or a mental health triage scale (67)	*System Performance*: no change in admission rates (79), ED LoS (63, 79), recognition of patient acuity (67) *Patient Outcomes*: decreased ED utilisation (62) *Staff Experience*: increased confidence (67)
**Discharge and transfer of care**	Refinement of discharge processes including default referral to psychiatry by medical staff (63) using observational beds to allow more time to collaborate with post-ED services (62) and communication processes (81) such as allocating a primary contact for psychiatry staff and patients (63), discharge checklists (81), and discharge appointments (81)	*System Performance*: decreased wait time (81), increased proportion discharged by 11 a.m (81)., reduced ED LoS (63) *Patient Outcomes*: reduced ED utilisation (62)
**Liaison Psychiatry Service**	Establishing a priority discussion for ED staff (i.e., nurse manager, social worker, or doctor) in the Liaison Psychiatry team morning shift handover meeting (63)	*System Performance*: reduced ED LoS (63)
**Telepsychiatry**	Telepsychiatry can include psychiatric assessments (64) and tele-mentoring consultations between interdisciplinary team members (e.g., psychiatrist, psychiatric assessment officer, behavioural health nurses, and ED staff) to provide ongoing support around the management of complex psychiatric patients (73, 82)	*System Performance*: consistent performance between face-to-face and telepsychiatry (64) interview duration reduced over time (64) *Patient Outcomes*: reduced ED utilisation (73) *Patient Experience*: preference for telepsychiatry if it reduces wait times (64) *Staff Experience*: no difference in preference between face-to-face and telepsychiatry (64), requires supporting infrastructure and straightforward processes (82)
**Role changes and rostering**	Role changes in the ED include clarification on legal obligations and safeguards regarding restraint and detention (63), the addition of psychiatric nurses in the ED (69, 80), a full-time psychiatrist and nurse practitioner employed in the ED (78), an onsite psychiatric assessment officer (to assess, brief intervention, and coordinate care) (73), or a mental health consultation liaison role (83) Rostering changes include the addition of a 10-h swing shift for ED residents with a later start time during the day to complement the 12-h regular shifts (65)	*System Performance*: reduced wait time (65, 78), reduced time to complete tasks (78), reduced ED LoS (63, 65), reduced admissions (69), reduced security staff standby hours (69), role utilised (83) *Patient Outcomes*: reduced ED utilisation (73), reduced LWBS *Patient Experience*: no change in patient satisfaction (80) *Staff Experience*: no effect on resident wellbeing or burnout (65), improvements in communication, collaboration, and timely care delivery (78), personalities were the main reason for success, though the psychiatric nurses felt isolated from the mental health team and out of touch with developments (80), role provides a resource to support better care delivery (83)
**Specialised unit within the ED**	Dedicated bed spaces within the ED such as a behavioural assessment unit (66), Psychiatric Assessment and Planning Units (75), psychiatric emergency service (85), and mental health short stay units (86) include beds for the assessment and management of patients (including behaviourally disturbed) in an environment designed to be safe and secure, allow close observation and provide timely access to specialist expertise and facilities for the appropriate use of sedation and restraint when required, irrespective of the patient’s primary diagnosis (66). These units also provide space for brief psychological support such as psychoeducation and safety planning, access to carer, consumer and family support, social worker input and liaison with community linkages (75, 76, 85, 86) Other dedicated spaces include designated interview rooms containing only reclining lounge chairs, staffed by two psychiatric RNs on shift (72)	*System Performance*: reduced wait times (76), reduced ED LoS (66, 76, 86), reduced use of restraint (66, 72, 76, 85), increased referrals (72), fewer code grey (66), increased completion of assessment (85), no change in pathology test use (85) *Patient Outcomes*: reduced involuntary commitment (72), no adverse events (75), no change in ED utilisation (85), reduced elopement *Patient Experience*: perceived as a sanctuary with caring and receptive staff (75) *Staff Experience*: ED staff felt assisted by PAPU but that it did no resolve flow issues (75)
**Education and training**	Formal training for triage nurses on mental health and illness (68)	*System Performance*: reduced ED LoS (68) *Staff Experience*: increased nurse confidence (68)
**Intervention teams**	Intervention teams can include different professions such as psychiatrists (70), nurses (70), nurse practitioners (84), administrative agents (70), clinicians (70), and family-peer support team members (70). The intervention teams provide psychosocial intervention (70), medication management (70), and referral (70). These teams are sometimes called brief intervention team (70), crisis centre team (70), family-peer support team (70), mental health liaison nurse (MHLN) team (84)	*System Performance*: reduced wait times (84), reduced ED LoS (84), increased referrals (84) *Patient Outcomes*: one near miss (84), few LWBS (84) *Patient Experience*: staff were compassionate and sensitive to them, that they listened carefully and genuinely helped (70). Patients also reported receiving rapid treatment; the treatment steps were explained, subsequent appointments set, and information on MH services made available (70), patients accept the model (84) *Staff Experience*: staff accept the model (84)
**Standardised protocol**	Standardisation of care through care protocols (71), COVID testing protocols (78), communication procedures (78), agitated behaviour management procedures (78), processes for managing medical stable intoxicated patients (78), standards of care for psychiatric evaluations (collecting information, documentation, and connecting with providers) (79), escalation processes with clear actions and reporting (86), refining the admission process to avoid ED assessments, local bed management rules (86), and can include training for the protocol (71) Changes to protocols also include the de-implementation of mandatory screening lab tests for psychiatric admission to the ordering of lab tests for patients being admitted to the inpatient psychiatry service “based on individual patient history and exam” (87)	*System Performance*: reduced time to task completion (78), no difference in admission rate (79), no change in ED LoS (79), reduced ED LoS (86, 87), reduced pathology test orders (87), reduced charges for orders (87) *Patient Outcomes*: no patient deaths (87) *Patient Experience*: no change in patient satisfaction (71), improved quality of information (71), increased patient knowledge (71), perceived coercion decreased during study period then increased during follow-up period (71), no change in ED utilisation *Staff Experience*: improvements in communication, collaboration, and timely care delivery (78)
**Environmental design**	Environmental design involves changes to the physical space based on behavioural design and psychological research such as the installation of a full-length glass, lockable door with a system that made it close automatically (74)	*System Performance*: reduced rates of seclusion and restraint (74)
**Case management**	Case management and care planning facilitates connections with appropriate community-based services (77), sometimes targeting frequent presenters and chronic and complex patients at risk of extended LoS (86)	*System Performance*: reduced ED LoS (86) *Patient Experience*: working relationships are important, service navigation is not easy, transition between service support is important, shame and stigma are barriers to engagement (77) *Staff Experience*: rapport is critical, case management is not a short-term relationship, service users have multiple existing connections that require significant coordination (77)

ED, emergency department; EMR, electronic medical record; LoS, length of stay; LWBS, left without being seen; PAPUs, Psychiatric Assessment and Planning Units; RN, registered nurse.

## 4 Discussion

We examined the research evidence provided by peer-reviewed literature describing strategies to improve ED care delivery for adults presenting with mental illness and measures of system performance, patient experience, patient outcomes, and staff experience. This systematic review found illness-specific strategies oriented to longer-term care delivery beyond the ED, and general mental illness interventions oriented to process improvements. Substance-related and addictive disorder interventions focused on the initiation of initial dose (e.g., buprenorphine or naloxone) with take home doses and clinic follow-up (42–45, 49–52). Similarly, strategies for suicide and deliberate self-harm presentations focused on assessment, care plan development and connecting with community-based support (53–55, 58–61). Strategies for mental illness presentations in general included modifications to the EMR to support decision making (62, 63, 67, 79), additional mental health roles (69, 73, 78, 80, 83) and intervention teams (70, 84), designated spaces for psychosocial assessment, treatment and referral (66, 72, 75, 76, 86), and refined discharge processes (62, 63, 81). For EDs and the communities they serve, considered selection of strategies and measures is essential in ensuring responsive, safe, and timely emergency care; however, without detailed descriptions of ED settings and use of common outcome measures, identification of high-impact interventions that might be transferable is challenging.

### 4.1 Identifying high-impact interventions for the local context

ED interventions interact with the characteristics, circumstances, and unique factors of the ED where they are implemented (88). Where an intervention was associated with favourable outcomes, contextual factors may have influenced these outcomes, but these were not consistently described across studies. More consistent reporting of interventions using reporting guidelines, such as the Template for Intervention Description and Replication (TIDieR) checklist (89), would be helpful in future research and for the overall development of the field. It was also challenging to judge unvalidated patient satisfaction surveys, particularly as patient characteristics such as education level and age have been associated with higher patient satisfaction scores (90). More consistent reporting of patient outcome measures, such as those advanced by the International Consortium for Health Outcomes Measurement (91), may assist in better identifying replicable high-impact interventions. While presentation-specific and systems-based solutions have the potential to improve the capacity of ED staff to provide care safely and ethically for adults presenting with mental illness, interventions must be aligned with current clinical guidelines and the purpose of the ED system. The evidence currently supporting the effectiveness of these strategies is limited with more detailed development of strategies and analysis needed to make meaningful progress in improving care delivery.

### 4.2 Evaluating the impact of interventions

Differences between the communities each ED served were reflected in the diverse selection of strategies and the measures employed to understand their effect. The importance of a comprehensive understanding of the effect of strategies on all of system performance, patient outcomes, patient experience, and staff experience, and the outcomes valued or prioritised locally was identified in this review. Only one of the implemented strategies, the ED-initiated MOUD model of care, reported outcomes across all four domains, accrued through eight separate studies. Furthermore, the outcomes reported by Sinclair et al. (80), Mitchell et al. (75), and Woo et al. (85) suggest that improvements in one domain, such as system performance, may not always translate to improvements in others, for example, patient and staff experience. For example, Sinclair measured an increase in the number of patients assessed but no difference in patient satisfaction, potentially because there was no difference in waiting time (a system performance measure that may be valued more highly by patients). Furthermore, Sinclair reported that while ED staff appreciated the addition of a specialist mental health nurse, the mental health staff integrated in the ED felt isolated from the mental health team and out of touch with developments in their specialty. Therefore, it is crucial to understand system performance goals as well as what patients and staff value, and evaluate interventions across all four domains to support the sustainability of improvement efforts.

### 4.3 Implications for clinical practice, policy, and research

The characteristics of the strategies and their evaluated outcomes suggest that adults are seeking ED care for mental illness that EDs are not resourced to provide. In attempting to bridge the care gap, EDs are often having to implement practices outside their operational and structural role of rapid assessment, stabilisation, and referral to hospital inpatient or community-based care, as one component of an integrated health system. This means that some of the reviewed strategies to improve care are not aligned with the ED system purpose, potentially exhausting the EDs’ capacity to respond to all patients’ needs effectively (92) and potentially diminishing the resilience of the ED system (93). Nevertheless, the ED is an important component of the care continuum and must therefore be integrated into the health system that cares for adults with mental illness, often in conjunction with comorbid, physical health issues. In this review, Consultation Liaison Psychiatry (CLP) in ED was found to improve system performance, patient outcomes, and patient experience (i.e., Opmeer et al. and Xanthopoulou et al.) (60, 61). CLP is a subspeciality of adult psychiatry that provides specialist medical expertise of the management of conditions occurring in areas overlapping mental and physical health (94). CLP has developed *ad hoc* over the last 20 years to meet needs, and has been variably funded with or without nursing or allied health representation (95). A 2023 review of CLP in 129 Australian hospitals found that the CLP interventions were all under-resourced in relation to need (94). Development of CLP as an appropriately resourced subspecialty may build capacity among existing non-mental health workforce and contribute to better outcomes and experiences for patients and staff, and better system performance. We echo Evans et al.’s (2018) recommendation for more rigorous evaluation of CLP models in ED using standardised outcomes. Going further, we emphasise the importance of describing interventions consistently, and measuring outcomes across all four domains of system performance, patient outcomes, and patient and staff experience.

### 4.4 Strengths and limitations

A comprehensive search and review process was used to identify and appraise empirical studies reporting strategies to improve care delivery for adults presenting to ED with mental illness. Limitations of the current review include our pragmatic choice to only include strategies implemented within the ED itself. As such, interventions outside of the ED including clinics that connect individuals with psychosocial support (e.g., agile psychological medicine clinics (96)) were not included, though these may impact care delivery in the ED. To align with legal frameworks in Australia, interventions for people with disability such as neurocognitive impairment were not included in this review. Patient and public were not involved in this review who may have contributed valuable insights into the experiences and outcomes of interest. We also chose to only include articles published in English, omitting potentially useful reports in other languages.

Owing to the volume of references identified by the search strategies and our aim to capture measures of effectiveness, we made a pragmatic decision to only include empirical studies and not include a grey literature review, hand searching, or subject matter expert consultation. As such, implemented but unpublished interventions were not included, which may have also contributed important information. Most studies did not report clear information regarding patient characteristics and intervention details; nor were the evaluation measures comprehensive. The nature of pragmatic naturalistic study designs may also introduce bias: allocation concealment was not used in two out of three randomised controlled trial (RCT) studies, and blinding did not occur or was not possible in two studies; most studies were quasi-experimental or non-randomised studies—participants in comparisons were not always similar, or it was unclear if participants were similar in 16 of 29 (55%) studies. The hospital context may also introduce bias: the type of hospital was not reported for 18 of 46 (39%) studies, and access to psychiatric services was not reported in 21 of 46 (45%) studies; most studies were conducted in urban settings, with only 4 of 46 (8%) including a rural site. Consequently, it was not possible to identify the key elements of interventions and features of ED environments that influence strategy evaluation measures. As a result, it is unclear what interventions are successful for whom, or if interventions result in negative impacts on patient outcomes, experiences, and staff experience.

### 4.5 Conclusion

We identified strategies for improving ED care delivery for mental illness presentations. The strategies included models of care (e.g., ED-initiated MOUD, ED-initiated social support, and deliberate self-harm), decision support tools, discharge and transfer refinements, case management, adjustments to liaison psychiatry services, telepsychiatry, changes to roles and rostering, environmental changes (e.g., specialised units within the ED), education, new multidisciplinary teams, and standardisations of care (e.g., assessment and monitoring). No single study evaluated all four domains of system performance, patient outcomes, patient experience, and staff experience. Furthermore, many strategies fill a gap in service delivery for patients that does not align with the functional purpose of the ED. The expanded scope of care delivered by EDs puts the system under considerable strain. We need to think critically about whether care is delivered in the right place at the right time for adults with mental illness. This would include developing capacity in community services as well as appropriately resourced CLP to support the ED to fulfil its role in delivering safe and timely urgent care.

## Author contributions

EA: Conceptualization, Data curation, Formal analysis, Investigation, Methodology, Project administration, Supervision, Validation, Visualization, Writing – original draft, Writing – review & editing. CC: Conceptualization, Data curation, Formal analysis, Investigation, Methodology, Project administration, Supervision, Validation, Visualization, Writing – original draft, Writing – review & editing. LR: Methodology, Project administration, Validation, Writing – review & editing, Conceptualization, Data curation, Investigation. LT: Conceptualization, Data curation, Investigation, Methodology, Validation, Writing – review & editing. AD: Conceptualization, Data curation, Investigation, Methodology, Writing – review & editing. JL: Conceptualization, Data curation, Investigation, Methodology, Writing – review & editing. AC: Conceptualization, Data curation, Investigation, Methodology, Writing – review & editing. LE: Conceptualization, Data curation, Investigation, Methodology, Writing – review & editing. AN: Conceptualization, Data curation, Investigation, Methodology, Writing – review & editing. MM: Conceptualization, Data curation, Investigation, Methodology, Writing – review & editing. KS: Conceptualization, Data curation, Investigation, Methodology, Writing – review & editing. DG: Conceptualization, Data curation, Investigation, Methodology, Writing – review & editing. RC-W: Conceptualization, Data curation, Funding acquisition, Investigation, Methodology, Resources, Writing – review & editing.
