# Supplementary material for: Improving emergency department care for adults presenting with mental illness: a systematic review of strategies and their impact on outcomes, experience, and performance
Source: Front Psychiatry. 2024 Feb 29;15:1368129. doi: 10.3389/fpsyt.2024.1368129 (PMC10937575; doi:10.3389/fpsyt.2024.1368129)
Supplement: Supplementary file 1 [file Table_1.docx]

Supplementary Material A

# Search Strategy

| **Database: Elsevier Scopus** via Macquarie University Library |
| --- |
| **SUBJECT #1: Emergency Department**  **KEYWORDS:** ( TITLE-ABS-KEY ( ( "hospital emergency service*" OR "emergency department*" OR "emergency unit*" OR "emergency ward*" OR "emergency room" * ) ) )  **MESH/ SUBJECT HEADING :** not applicable for Elsevier Scopus |
| **SUBJECT #2: Improvement**  **KEYWORDS** ( TITLE-ABS-KEY ( "quality improve*" OR "quality performance" OR "quality intervention" OR "care improve*" OR "quality assess*" OR "quality assurance" OR "psyc* assessment" OR "nursing assessment" OR "case management" OR "patient care planning" OR "discharge planning" OR "multidisciplinary intervention" OR "improv* outcomes") )  **MESH/ SUBJECT HEADING :** not applicable for Elsevier Scopus |
| **SUBJECT #3 Outcomes**  **Keywords:** TITLE-ABS-KEY ( "Clinical Outcome*" OR ( patient W/2 outcome* ) OR ( consumer W/2 outcome* ) OR "health outcomes" OR reattendance OR representation OR readmission OR "re-attendance" OR "adverse events" OR "clinical errors" OR "medication error" OR "missed diagnosis" OR "mortality" OR "morbidity" OR "pain" OR "quality of life" OR "HRQoL" OR "health related quality of Life" OR "patient satisfaction" OR "patient experience" OR ( patient W/2 ( experience OR perspective OR opinion OR perception OR view OR feedback OR preference ) ) OR "staff experience" OR "job satisfaction" OR "intention to stay" OR "work environment" OR "employee complaint ratio" OR "patient wait time satisfaction" OR "patient reported problems" OR "patient reported outcome" OR "patient reported experience" OR "patient complaints" OR "left before treatment complete" OR "LAMA" OR "left against medical advice" OR "LWBS" OR "left without being seen" OR "LBTC" OR "left without Treatment complete" OR "PWLBST" OR "Patients who left before supposed to" OR "PREM" OR "patient reported experience measure" OR "ED performance" OR "performance indicator" OR "performance measure" OR "benchmark" OR "length of stay" OR los OR "functional decline" OR "hospital admission" OR "admission rates" OR hospitalization OR hospitalisation OR recidivism OR "service use rates" OR "health care quality" OR "satisfaction with care" OR "caregiver outcome" )  **MESH/ SUBJECT HEADING :** not applicable for Elsevier Scopus |
| **SUBJECT #4: Mental Health**  **KEYWORDS:** TITLE-ABS-KEY ("mental health" OR “mental illness” OR “mental disorder*” OR “psychiatr*”)  **MESH/ SUBJECT HEADING :** not applicable for Elsevier Scopus |
| **LIMITATIONS:**  AND NOT ( TITLE-ABS-KEY ( pediatric OR child* OR adolescent OR ambulatory OR outpatient) |
| **Database: OVID EMBASE** via Macquarie University Library |
| **SUBJECT #1: Emergency Department**  **KEYWORDS:** ("hospital emergency service*" or "hospital emergency service*" or "emergency department*" or "emergency unit" or "emergency ward*" or "emergency room*").ti,ab  **EMTREE SUBJECT HEADING :** emergency ward/ or hospital emergency service/ or emergency health service/ |
| **SUBJECT #2: Improvement**  **KEYWORDS** ("quality improve*" OR "quality performance" OR "quality intervention" OR "care improve*" OR "quality assess*" OR "quality assurance" OR "psyc* assessment" OR "nursing assessment" OR "case management" OR "patient care planning" OR "discharge planning" OR "multidisciplinary intervention" OR "improv* outcomes").ti,ab  **EMTREE SUBJECT HEADING :** total quality management/ or Case Management/ or Nursing Assessment/ or health risk assessment/ |
| **SUBJECT #3 Outcomes**  **Keywords:** ( "Clinical Outcome*" OR ( patient AND outcome ) OR ( consumer AND outcome ) OR "health outcomes" OR reattendance OR representation OR readmission OR "re-attendance" OR "adverse events" OR "clinical errors" OR "medication error" OR "missed diagnosis" OR "mortality" OR "morbidity" OR "pain" OR "quality of life" OR "HRQoL" OR "health related quality of Life" OR "patient satisfaction" OR "patient experience" OR ( patient ADJ2 ( experience OR perspective OR opinion OR perception OR view OR feedback OR preference ) ) OR "staff experience" "staff experience" OR "job satisfaction" OR "intention to stay" OR "work environment" OR "employee complaint ratio" OR "patient wait time satisfaction" OR "patient reported problems" OR "patient reported outcome" OR "patient reported experience" OR "patient complaints" OR "left before treatment complete" OR "LAMA" OR "left against medical advice" OR "LWBS" OR "left without being seen" OR "LBTC" OR "left without Treatment complete" OR "PWLBST" OR "Patients who left before supposed to" OR "PREM" OR "patient reported experience measure" OR "ED performance" OR "performance indicator" OR "performance measure" OR "benchmark" OR "length of stay" OR LOS OR "functional decline" OR "hospital admission" OR "admission rates" OR hospitalization OR hospitalisation OR recidivism OR "service use rates" OR "health care quality" OR "satisfaction with care" OR "caregiver outcome").ti,ab  **EMTREE SUBJECT HEADING :** treatment outcome/ or adverse outcome/ or hospital readmission/ or medical error/ or mortality / or morbidity/or pain/ or quality of life/ or patient preference/ or patient-reported outcome/ or performance indicator/ or health care quality/ or benchmarking/ or patient satisfaction/ or daily life activity/ or outcome assessment/ or patient referral/ or consultation/ or hospital admission/ or "length of stay"/ |
| **SUBJECT #4: Mental Health**  **KEYWORDS:**  ("mental health" or "mental illness" or " psychiatr*" or "mental disorder*").ti,ab.  **MESH/ SUBJECT HEADING : exp** mental disease |
| **LIMITATONS:**  not (pediatric or child*).ti,ab |
| **Database: EBSCOhost CINAHL Complete** via Macquarie University Library |
| **SUBJECT #1: Emergency Department**  **KEYWORDS:**  TI ( "emergency service*" OR "hospital emergency service*" OR "emergency department*" OR "emergency unit" OR "emergency ward*" OR "emergency room*" )  OR AB ( "emergency service*" OR "hospital emergency service*" OR "emergency department*" OR "emergency unit" OR "emergency ward*" OR "emergency room*" ) **MESH/ SUBJECT HEADING :** MH ("Emergency Medical Services+" OR "Emergency Service+” OR "Acute Care+") |
| **SUBJECT #2: Improvement**  **KEYWORDS**  TI ( "quality improve*" OR "quality performance" OR "quality intervention" OR "care improve*" OR "quality assess*" OR "quality assurance" ) OR AB ( "quality improve*" OR "quality performance" OR "quality intervention" OR "care improve*" OR "quality assess*" OR "quality assurance" OR "psyc* assessment" OR "nursing assessment" OR "case management" OR "patient care planning" OR "discharge planning" OR "multidisciplinary intervention" OR "improv* outcomes" )  **MESH/ SUBJECT HEADING :** (MH "Quality Improvement+") OR (MH "Evaluation and Quality Improvement Program") OR (MH "Quality Management, Organizational") OR (MH "Case Management") OR (MH "Nursing Assessment") OR (MH "Risk Assessment") |
| **SUBJECT #3 Outcomes**  **Keywords:** TI ( ( "Clinical Outcome*" OR ( patient N2 outcome ) OR ( consumer N2 outcome ) OR "health outcomes" OR reattendance OR representation OR readmission OR "re-attendance" OR "adverse events" OR "clinical errors" OR "medication error" OR "missed diagnosis" OR "mortality" OR "morbidity" OR "pain" OR "PROM*" OR "quality of life" OR "HRQoL" OR "health related quality of Life" OR "patient satisfaction" OR "patient experience" OR ( patient N2 ( experience OR perspective OR opinion OR perception OR view OR feedback OR preference ) ) OR "staff experience" OR "patient wait time satisfaction" OR "patient reported problems" OR "patient reported outcome" OR "patient reported experience" OR "patient complaints" OR "left before treatment complete" OR "LAMA" OR "left against medical advice" OR "LWBS" OR "left without being seen" OR "LBTC" OR "left without Treatment complete" OR "PWLBST" OR "Patients who left before supposed to" OR "PREM" OR "patient reported experience measure" OR "work environment" OR "employee complaint ratio" OR "ED performance" OR "performance indicator" OR "performance measure" OR "benchmark" OR "length of stay" OR LOS OR "functional decline" OR "hospital admission" OR "admission rates" OR hospitalization OR hospitalisation OR recidivism OR "service use rates" OR "health care quality" OR "satisfaction with care" OR "caregiver outcome" )  OR AB ( ( "Clinical Outcome*" OR ( patient N2 outcome ) OR ( consumer N2 outcome ) OR "health outcomes" OR reattendance OR representation OR readmission OR "re-attendance" OR "adverse events" OR "clinical errors" OR "medication error" OR "missed diagnosis" OR "mortality" OR "morbidity" OR "pain" OR "quality of life" OR "HRQoL" OR "health related quality of Life" OR "patient satisfaction" OR "patient experience" OR ( patient N2 ( experience OR perspective OR opinion OR perception OR view OR feedback OR preference ) ) OR "staff experience" OR "job satisfaction" OR "intention to stay" OR "work environment" OR "employee complaint ratio" OR "patient wait time satisfaction" OR "patient reported problems" OR "patient reported outcome" OR "patient reported experience" OR "patient complaints" OR "left before treatment complete" OR "LAMA" OR "left against medical advice" OR "LWBS" OR "left without being seen" OR "LBTC" OR "left without Treatment complete" OR "PWLBST" OR "Patients who left before supposed to" OR "PREM" OR "patient reported experience measure" OR "ED performance" OR "performance indicator" OR "performance measure" OR "benchmark" OR "length of stay" OR LOS OR "functional decline" OR "hospital admission" OR "admission rates" OR hospitalization OR hospitalisation OR recidivism OR "service use rates" OR "health care quality" OR "satisfaction with care" OR "caregiver outcome")  **MESH/ SUBJECT HEADING :** (MH "Treatment Outcomes+") OR (MH "Readmission") OR (MH "Health Care Errors") OR (MH "Mortality") OR (MH "Morbidity") OR (MH "Pain") OR (MH "PROM") OR (MH "Qualtiy of Life") OR (MH "Patient Preference") OR (MH "Patient-Reported Outcomes") OR (MH "Work environment") OR (MH "Quality of Health Care") OR (MH "Benchmarking") OR (MH "Process Assessment (Health Care)") OR (MH "Patient Satisfaction+") OR (MH "Activities of Daily Living+") OR (MH "Outcome Assessment") OR (MH "Patient Admission/SN") OR (MH "Length of Stay") OR (MH "Continuity of Patient Care+") |
| **SUBJECT #4: Mental Health**  **KEYWORDS:** TI ("mental health" OR "mental illness" OR “mental disorder*” OR "psyc*")  OR AB ("mental health" OR "mental illness" OR " psychiatr*" OR “mental disorder*”)  **MESH/ SUBJECT HEADING :** MH ("mental disorder*") |
| **Filter:**  NOT ((TI (pediatric OR child*)) OR (AB (pediatric OR child*))) |
| **Database: Ovid MEDLINE** via Macquarie University Library |
| **SUBJECT #1: Emergency Department**  **KEYWORDS:** ("hospital emergency service*" or "hospital emergency service*" or "emergency department*" or "emergency unit" or "emergency ward*" or "emergency room*").ti,ab  **MESH/ SUBJECT HEADING :** Emergency Service, Hospital/ or Emergency Medical Service/ |
| **SUBJECT #2: Improvement**  **KEYWORDS** ("quality improve*" OR "quality performance" OR "quality intervention" OR "care improve*" OR "quality assess*" OR "quality assurance" OR "psyc* assessment" OR "nursing assessment" OR "case management" OR "patient care planning" OR "discharge planning" OR "multidisciplinary intervention" OR "improv* outcomes").ti,ab  **MESH/ SUBJECT HEADING:** Quality Improvement/ |
| **SUBJECT #3 Outcomes**  **Keywords:** ( "Clinical Outcome*" OR ( patient AND outcome ) OR ( consumer AND outcome ) OR "health outcomes" OR reattendance OR representation OR readmission OR "re-attendance" OR "adverse events" OR "clinical errors" OR "medication error" OR "missed diagnosis" OR "mortality" OR "morbidity" OR "pain" OR "quality of life" OR "HRQoL" OR "health related quality of Life" OR "patient satisfaction" OR "patient experience" OR ( patient ADJ2 ( experience OR perspective OR opinion OR perception OR view OR feedback OR preference ) ) OR "staff experience" OR "job satisfaction" OR "intention to stay" OR "work environment" OR "employee complaint ratio" OR "patient wait time satisfaction" OR "patient reported problems" OR "patient reported outcome" OR "patient reported experience" OR "patient complaint*" OR "left before treatment complete" OR "LAMA" OR "left against medical advice" OR "LWBS" OR "left without being seen" OR "LBTC" OR "left without Treatment complete" OR "PWLBST" OR "Patients who left before supposed to" OR "PREM" OR "patient reported experience measure" OR "ED performance" OR "performance indicator" OR "performance measure" OR "benchmark" OR "patient satisfaction" OR "length of stay" OR "LOS" OR "functional decline" OR "hospital admission" OR "admission rates" OR hospitalization OR hospitalisation OR recidivism OR "service use rates" OR "health care quality" OR "satisfaction with care" OR "caregiver outcome").ti,ab  **MESH/ SUBJECT HEADING :** Treatment Outcome/ or Patient Reported Outcome Measures/ or "Patient Readmission"/ or Medical Errors/ or Mortality/ or Morbidity/ or Pain/ or "Quality of Life"/ or Patient Preference/ or Benchmarking/ or Patient Satisfaction/ or "Activities of Daily Living"/ or Outcome Assessment, Health Care/ or "Referral and Consultation"/ or Patient Admission/ or "Length of Stay"/ or "Continuity of Patient Care"/ |
| **SUBJECT #4: Mental Health**  **KEYWORDS:** ("mental health" or "mental illness" or " psychiatr*" or “mental disorder*”).ti,ab  **MESH/ SUBJECT HEADING :** exp mental disorders |
| **LIMITATIONS:**  not (pediatric or child* or ambulatory or outpatient).ti,ab |
