# Supplementary material for: Improving emergency department care for adults presenting with mental illness: a systematic review of strategies and their impact on outcomes, experience, and performance
Source: Front Psychiatry. 2024 Feb 29;15:1368129. doi: 10.3389/fpsyt.2024.1368129 (PMC10937575; doi:10.3389/fpsyt.2024.1368129)
Supplement: Supplementary file 2 [file Table_2.docx]

Supplementary Material B

# Data extraction form

| **General Information** |
| --- |
| **Title**  Title of paper / abstract / report that data are extracted from *(free-text response)* |
| **Lead author**  e.g., Austin, E. *(free-text response)* |
| **Year** *(free-text response)* |
| **Study aim**  Copy and paste from the article. *(free-text response)* |
| **Country in which the study conducted**   - United States - UK (England, Scotland, Wales, Northern Ireland) - Canada - Australia - Ireland - Other   *(Check-box response)* |
| **Notes**  If not mentioned explicitly, report the first author location *(free-text response)* |
| **Characteristics of included studies** |
| **Methods** |
| **Study design**  The study design you select determines the Critical Appraisal tool you use. See parentheses for a brief study design description and the relevant critical appraisal tool to be used for this study design.   - RCT (randomized allocation of participants to control/ intervention groups; RCT) - Quasi-experimental (no random assignment of participants to a group; QUASI-EXPERIMENTAL) - Pre-post (before and after study; outcomes are measured before intervention and then again after; QUASI-EXPERIMENTAL) - Time series (specific analysis of a sequence of data points over an interval of time; e.g., trends; QUASI-EXPERIMENTAL) - Case-control (a type of observational study; cases have the outcome of interest; used to look at factors associated with diseases or outcomes; CASE-CONTROL) - Cohort (a type of longitudinal study; retrospective; participants share a common characteristic; COHORT) - Cross-sectional (a type of observational study; outcome and exposure are measured at the same time; e.g., survey; CROSS-SECTIONAL) - Descriptive (describes the distribution of outcomes; NO CRITICAL APPRAISAL REQUIRED) - Qualitative (non-numerical data to understand problem, concepts, opinion, experiences; QUALITATIVE) - Other   *(Check-box response)* |
| **Primary aim of the intervention**  (NOT the aim of the study) *(free-text response)* |
| **Secondary aims**  Other aims of the intervention  (Not the aim of the study) *(free-text response)* |
| **Total number of EDs included in the study** |
| **Description of the intervention**  Please copy and paste a description of the intervention implemented. *(free-text response)* |
| **Participants** |
| **Total number of participants in the study** *(free-text response)* |
| **Total number of participants in the intervention** *(free-text response)* |
| **Total number of participants in the control**  If applicable. *(free-text response)* |
| **Mental health condition focus**  Select all that apply.  Use the text box to report "other' mental health focus   - Depression - Anxiety - Suicidal / self-harm - Substance related and addictive disorders (e.g., Substance use disorder) - Bipolar - Trauma and stressor related - Eating disorders - Other   *(Check-box response)* |
| **Participant inclusion criteria**  If more than one inclusion criteria please use a semicolon (;) between the different criteria. *(free-text response)* |
| **Participant exclusion criteria**  If more than one exclusion criteria please use a semicolon (;) between the different criteria. *(free-text response)* |
| **Results** |
| **Intervention participants**  -Report age (min, max, mean and SD) to 1 decimal point when reported by authors.  -Report % to 1 decimal point when reported by authors. When % is reported without a decimal point, enter % without a decimal point.  -When there is more than 1 intervention group, use the additional group row  *In a pre-post study, the post group are considered the intervention group   \|  \| Intervention group name \| Min Age \| Max Age \| Mean \| Standard Deviation \| Man/ male [n,%] \| Woman/ female [n,%] \| Non-binary [n,%] \| Other [n,%] \| \| --- \| --- \| --- \| --- \| --- \| --- \| --- \| --- \| --- \| --- \| \| Group 1 \|  \|  \|  \|  \|  \|  \|  \|  \|  \| \| Group 2 \|  \|  \|  \|  \|  \|  \|  \|  \|  \|   *(table completion response)* |
| **Control participants**  Report to 1 decimal point when reported by authors.  *In a pre-post study, the pre-group are considered the control group   \|  \| Intervention group name \| Min Age \| Max Age \| Mean \| Standard Deviation \| Man/ male [n,%] \| Woman/ female [n,%] \| Non-binary [n,%] \| Other [n,%] \| \| --- \| --- \| --- \| --- \| --- \| --- \| --- \| --- \| --- \| --- \| \| Group 1 \|  \|  \|  \|  \|  \|  \|  \|  \|  \| \| Group 2 \|  \|  \|  \|  \|  \|  \|  \|  \|  \|   *(table completion response)* |
| **Evaluated outcomes**  Select all that apply.  Examples are not comprehensive.   - System performance (e.g., Waiting time, LoS, time to treat, admissions, errors) - Patient outcomes (e.g., re-admission, adverse event, medical errors, missing diagnosis, mortality, pain, quality of life) - Patient experience (e.g., experience, complaints, did not wait, left without being seen, left at own risk) - Staff experience (e.g., experience, job satisfaction, intention to stay)   *(Check-box response)* |
| **Outcome table (Time Point 1)**  -Fill in outcome measure and add unit of measurement in parenthesis e.g., ED LOS (hours), mortality (7-day).  -Extract data for outcome under relevant column.  -Report to two decimal places except for:  a) Report % to 1 decimal point when reported by authors. When % is reported without a decimal point, enter % without a decimal point.  b) Report p-values to 3 decimal places. Include < and > where appropriate.  Please add a note in the relevant box of any deviations. More information is better than less.   \|  \| Outcome measure \| Con n (%) \| Int n (%) \| Con mean (SD) \| Int mean (SD) \| Con median (IQR) \| Int median (IQR \| Con OR (95% CI) \| Int OR (95% CI) \| Con IRR (95% CI) \| Int IRR (95% CI) \| Con RR (95% CI) \| Int RR (95% CI) \| P Value \| \| --- \| --- \| --- \| --- \| --- \| --- \| --- \| --- \| --- \| --- \| --- \| --- \| --- \| --- \| --- \| \| Outcome measure 1 \|  \|  \|  \|  \|  \|  \|  \|  \|  \|  \|  \|  \|  \|  \| \| Outcome measure 2 \|  \|  \|  \|  \|  \|  \|  \|  \|  \|  \|  \|  \|  \|  \| \| Outcome measure 3 \|  \|  \|  \|  \|  \|  \|  \|  \|  \|  \|  \|  \|  \|  \| \| Outcome measure 4 \|  \|  \|  \|  \|  \|  \|  \|  \|  \|  \|  \|  \|  \|  \| \| Outcome measure 5 \|  \|  \|  \|  \|  \|  \|  \|  \|  \|  \|  \|  \|  \|  \| \| Outcome measure 6 \|  \|  \|  \|  \|  \|  \|  \|  \|  \|  \|  \|  \|  \|  \| \| Outcome measure 7 \|  \|  \|  \|  \|  \|  \|  \|  \|  \|  \|  \|  \|  \|  \| \| Outcome measure 8 \|  \|  \|  \|  \|  \|  \|  \|  \|  \|  \|  \|  \|  \|  \| \| Outcome measure 9 \|  \|  \|  \|  \|  \|  \|  \|  \|  \|  \|  \|  \|  \|  \| \| Outcome measure 10 \|  \|  \|  \|  \|  \|  \|  \|  \|  \|  \|  \|  \|  \|  \|   Note. Con, Control; Int, Intervention  *(table completion response)* |
| **Outcome table (Time Point 2)**   \|  \| Outcome measure \| Con n (%) \| Int n (%) \| Con mean (SD) \| Int mean (SD) \| Con median (IQR) \| Int median (IQR \| Con OR (95% CI) \| Int OR (95% CI) \| Con IRR (95% CI) \| Int IRR (95% CI) \| Con RR (95% CI) \| Int RR (95% CI) \| P Value \| \| --- \| --- \| --- \| --- \| --- \| --- \| --- \| --- \| --- \| --- \| --- \| --- \| --- \| --- \| --- \| \| Outcome measure 1 \|  \|  \|  \|  \|  \|  \|  \|  \|  \|  \|  \|  \|  \|  \| \| Outcome measure 2 \|  \|  \|  \|  \|  \|  \|  \|  \|  \|  \|  \|  \|  \|  \| \| Outcome measure 3 \|  \|  \|  \|  \|  \|  \|  \|  \|  \|  \|  \|  \|  \|  \| \| Outcome measure 4 \|  \|  \|  \|  \|  \|  \|  \|  \|  \|  \|  \|  \|  \|  \| \| Outcome measure 5 \|  \|  \|  \|  \|  \|  \|  \|  \|  \|  \|  \|  \|  \|  \| \| Outcome measure 6 \|  \|  \|  \|  \|  \|  \|  \|  \|  \|  \|  \|  \|  \|  \| \| Outcome measure 7 \|  \|  \|  \|  \|  \|  \|  \|  \|  \|  \|  \|  \|  \|  \| \| Outcome measure 8 \|  \|  \|  \|  \|  \|  \|  \|  \|  \|  \|  \|  \|  \|  \| \| Outcome measure 9 \|  \|  \|  \|  \|  \|  \|  \|  \|  \|  \|  \|  \|  \|  \| \| Outcome measure 10 \|  \|  \|  \|  \|  \|  \|  \|  \|  \|  \|  \|  \|  \|  \|   Note. Con, Control; Int, Intervention  *(table completion response)* |
| **Limitations**  Limitations identified by the authors *(free-text response)* |
| **Comments**  Please provide any information here from the article that did not fit into the above response areas. *(free-text response)* |
