# Supplementary material for: Improving emergency department care for adults presenting with mental illness: a systematic review of strategies and their impact on outcomes, experience, and performance
Source: Front Psychiatry. 2024 Feb 29;15:1368129. doi: 10.3389/fpsyt.2024.1368129 (PMC10937575; doi:10.3389/fpsyt.2024.1368129)
Supplement: Supplementary file 3 [file Table_3.docx]

Supplementary Material C

Table 1. Characteristics of the included studies

| **Author, Year, Country** | **Type of study** | **Aim** | **Period of Study** | **Number of participants** | **Intervention Type** | **Outcome Assessed** | **Effect** |
| --- | --- | --- | --- | --- | --- | --- | --- |
| **Substance use and addictive disorder strategies** | | | | | | | |
| Bogan, 2020, USA [43] | Descriptive | This pilot program aimed to foundationally integrate universal Screening, Brief Interventions and Referral to Treatment (SBIRT) in hospital EDs to identify patients with at-risk substance use | December 2017 – March 2019 | 3 EDs: a large academic medical center, a large private hospital, and a small  community hospital; 2270 patients | 1. ED-initiated MOUD model of care  2. Decision support tools  3. Discharge and transfer of care | **System Performance:** eligibility of Buprenorphine; attendance at follow-up appointment, 30 day treatment adherence | **System Performance:** 241 patients of the 535 eligible received buprenorphine; 78% (187) attended the follow-up appointment, 59% of the 187 remained in treatment 30-days later |
| Butler, 2022, USA [44] | Pre-post (Quasi-experimental) | To evaluate effectiveness of ED-initiated buprenorphine treatment pathways on prescribed opioid use disorder (OUD)-related treatments | July 1, 2018 – June 30, 2019 | 1 ED; 769 patients | ED-initiated MOUD model of care | **System Performance:** number of OUD-related treatments | **System Performance:** 50% increase (1.66 additional) in OUD treatments per day |
| Faude, 2023, USA [45] | Quasi-experimental | To increase treatment engagement with Medications for Opioid Use Disorder (MOUD) at 30 days compared with referral alone. | December 2018 - July 2020 | 3 EDs, 1339 patients, 28 clinicians | ED-initiated MOUD model of care | **System Performance:** MOUD administered in the ED, ED LoS  **Patient outcomes:** ED revisit within 30 days, hospital readmission  **Staff Experience:** staff experience | **System Performance:** 23% received MOUD in the ED, 5.3 hours ED LoS  **Patient outcomes:** 35% ED revisit within 30 days, 6.5% hospital readmission  **Staff Experience:** clinicians varied in confidence, patient readiness, and perceptions of receptivity to treatment |
| Gertner, 2021, USA [46] | Quasi-experimental | To provide ED-initiated buprenorphine utilising a universal screening approach combined with a brief intervention delivered by a Peer Support Specialist in a large ED | January - June 2019 | 1 ED, 1037 patients screened | ED-initiated MOUD model of care | **System Performance:** number of people screened, number screened positive and requested treatment, number received MOUD in ED  **Patient Outcome:** number who attended intake appointment | **System Performance:** 1,037 patients screened, 23 screened positive and requested treatment, 7 received MOUD in the ED  **Patient Outcomes:** 4 attended intake appointment |
| Kahler, 2017, USA [51] | Pre-post (Quasi-experimental) | To evaluate the effectiveness of a chronic pain superuser opioid-seeking transition protocol on ED visits, opioid prescriptions, ECGs, pathology and radiology tests | Not reported | 1 ED, 243 patients with 12 months of data pre and post intervention | Transfer of care and patient discharge | **System Performance:** number of ED visits/ year, opioid prescriptions/ year, ECGs, pathology and radiology tests  **Patient Outcome:** number of: hospitalisations/ year, hospital days/ year, clinic visits/ year | **System Performance:** 58% decrease in number of ED visits/ year, 30% decrease in opioid prescriptions/ year, 50% decrease in ECGs, 46% decrease in pathology and 44% decrease in radiology tests  **Patient Outcome:** decrease in hospitalisations/ year, 85% decrease in hospital days/ year, 13% decrease in clinic visits/ year |
| Lowenstein, 2023, USA [50] | Quasi-experimental | Evaluate effectiveness of a triage protocol to increase greater increase in withdrawal assessment, buprenorphine prescriptions, and naloxone prescriptions | January 2020 - June 2022 | Three EDs between March and July 2021 constituted the intervention group (n=2462 patients), and the remaining 2 EDs served as controls (n=731 patients) | Decision support tools | **System Performance:** withdrawal assessment, prescriptions, Clinical Opioid Withdrawal Scale (COWS) measured, medications for MOUD, medications at discharge | **System Performance:** withdrawal assessment identified 2-3 patients per day, prescriptions, increased number of COWS measured, medications at discharge increased in intervention EDs |
| Lukacs, 2023, USA [52] | Pre-post (Quasi-experimental) | To describe the impact of Peer Recovery Coach (PRC) engagement during an ED visit on subsequent emergency department utilization | September 1, 2018 (the onset of the program) to September 30, 2020 | 3 EDs; 448 patients who completed an initial engagement encounter | ED-initiated social support model of care | **Patient Outcomes:** ED utilisation, engagement with resources, reported abstinence | **Patient Outcomes:** decreased ED utilisation, particularly for those with more frequent ED visits prior to PRC, 61% - 75% reported engagement with resources, 40%-64% reported abstinence at 90 days |
| McCormack, 2023, USA [47] | Descriptive | To offer ED patients with opioid use disorder medication-assisted therapy (ED-initiated buprenorphine) | September 2018 - April 2019 | 3 EDs; 2,522 patients screened positive, 38 completed final visit | ED-initiated MOUD model of care | **System Performance:** feasibility  **Patient Outcomes:** received ED-initiated medication, number engaged in formal treatment 30 after index visit, likelihood of overdose within 30 days, adverse events, mortality  **Patient Experience:** patient satisfaction  **Staff Experience:** readiness to provide ED-initiated medication | **System Performance:** required changes to resources, referrals, policy  **Patient Outcomes:** 112 received ED-initiated medication, 40 were estimated to be engaged in formal treatment 30 after index visit, decreased likelihood of overdose within 30 days, no adverse events or deaths  **Patient Experience:** patients were satisfied  **Staff Experience:** increase in readiness to provide ED-initiated medication |
| McLane, 2020, Canada [48] | Descriptive | Evaluate the effectiveness of an initiation and referral pathway on monthly ED prescribing rates of buprenorphine and naloxone | January 2018 - September 2018 | 3 EDs, recipients of medication (n=47), patients who did not receive medication (n=335) | ED-initiated MOUD model of care | **System Performance:** median ED LoS  **Patient Outcomes:** prescriptions filled (at 30 days, 60 days, 90days), referrals to clinics | **System Performance:** no difference in median ED LoS  **Patient Outcomes:** 72% of those discharged with medication filled prescriptions at 30 days, 60 days, 90 days; 43% of 37 patients attended clinic follow-up appointment |
| Murphy, 2023, USA [49] | Pre-post (Quasi-experimental) | Reduce daily alcohol consumption, improve quality of life; reduce alcohol-related life consequences; reduce WHO drinking risk level in individuals with alcohol use disorder | 12 weeks | 1 ED; 32 patients were enrolled | ED-initiated MOUD model of care | **Patient Outcomes:** alcohol use, drinking risk level, alcohol related life consequences, quality of life | **Patient Outcomes:** reduced alcohol use, reduced drinking risk level, improved quality of life |
| Solomon, 2023, USA [42] | Cohort | To evaluate the association between hospitals attesting to an ED buprenorphine treatment O-HQIP pathway and patients’ subsequent initiation of buprenorphine treatment | January 1, 2016 - December 31, 2020 | 1 ED; patients with OUD (n=17,428) | ED-initiated MOUD model of care | **Patient Outcomes:** prescription fills within 30 days of ED discharge | **Patient Outcomes:** 50% improvement in the rate of prescription fills within 30 days of ED discharge |
| **Suicide and self-harm strategies** | | | | | | | |
| Bryan, 2018, USA [57] | Randomised controlled trial | To lower suicide risk of acutely suicidal active-duty Army personnel (reported in another paper). To improve emotional state and lower inpatient admissions of acutely suicidal active-duty Army personnel. | Not reported | 1 ED, contract for safety (n=32), standard response plan (n=32), enhanced crisis response plan (n=33), Total = 97. | Self-harm model of care | **Patient Outcomes:** ten cognitive-affective states were assessed with the Visual Mood Analog Scale (VMAS) Scale for Suicide Ideation (SSI); Beck Hopelessness Scale; Beck Depression Inventory— II; PTSD Checklist, Military Version (PCL-M); and Suicide Attempt Self-Injury Interview | **Patient Outcomes:** both Crisis Response Plans (CRP; standard or enhanced) significantly reduced negative emotional states than the contract for safety, but the two CRP groups did not differ from each other |
| Clarke, 2002, UK [54] | Randomised controlled trial | To compare routine management enhanced by nurse-led case management with routine management only | February 1997 - March 1998 | 2 EDs, routine care (n=247), case management (n=220), total = 467. | Case management | **Patient Outcomes:**  one readmission to ED within 12-months, multiple readmissions to ED within 12-months | **Patient Outcomes:** no difference in one readmission, case management patients had a greater number of multiple readmissions. |
| Inui-Yukawa, 2021, Japan [53] | Randomised controlled trial | To prevent recurrent suicidal behaviour by suicide attempters in Japan | July 1, 2006, - December 31, 2009 | 17 EDs, intervention group (n=297), control group (n=295), total 592 | Case management | **Patient Outcomes:** number of recurrent suicide attempts (1 month, 3 months, 6 months, 12 months, 18 months), Overall self-harm I, Suicide attempts, Non-suicidal self-harm | **Patient Outcomes:** decrease in suicide attempts, decrease in self-harm |
| Kim, 2022, USA [60] | Pre-post (Quasi-experimental) | To evaluate the impact of an emergency psychiatric assessment, treatment, and healing (EmPATH) unit in the emergency department (ED) on hospital admissions, ED length of stay, and 30-day follow-up | Pre-implementation phase: November 15, 2017, - May 15, 2018, Post-implementation phase: November 15, 2018, to May 15, 2019 | 1 ED; Pre-implementation phase (n=435), post-implementation phase (n=527), total 962 patients | Specialised unit within the ED | **System Performance:** hospital admissions, ED LoS, restraint use,  **Patient Outcome** ED utilisation | **System Performance:** reduced hospital admissions, decreased ED LoS, no difference in restraint use  **Patient Outcome:** reduced ED utilitsation |
| Kroll, 2020, USA [61] | Descriptive | To determine whether continuous virtual monitoring (i.e., patient observation through video technology), can be used to monitor suicide risk in the general hospital and emergency department (ED) | June 2017 - March 2018 | 1 ED; patients on hospital floors (n=27; 69%), patients in the ED (n=12, 31%) in the ED, Total 39 patients | Suicide risk monitoring protocol | **System Performance:** monitoring hours, verbal interventions, stat alarms  **Patient Outcomes:** adverse events, no-harm incidents  **Staff Experience:** favourability, preference for virtual, preference for 1:1 | **System Performance:** 2,318 monitoring hours  **Patient Outcomes:** No adverse events, two no-harm incidents  **Staff Experience:** high favourability, preference for virtual, no difference in favourability |
| Lepping, 2006, UK [55] | Pre-post (Quasi-experimental) | To examine whether a self-harm pathway, developed by a local self-harm service planning group, could increase the number of psychosocial assessments | 2002 (June-July) – 2004 (June-August) | 1 ED; pre-intervention (n=335), post-intervention (n=388), total 723 | Self-harm model of care | **System Performance:** psychosocial assessments (requested, completed, performed within 24 hours), adherence to pathway | **System Performance:** increase in requested (57% to 85%), completed (47% to 70%) psychosocial assessments, assessments performed within 24 hours increased (59% to 92%), 71% of all forms were fully completed |
| Morgan, 2000, UK [56] | Pre-post (Quasi-experimental) | To increase the rate of referral for psychosocial assessment of individuals who presented at the department following acts of deliberate self-harm | Pre-intervention period: 1 March - 31 July 1996; post-intervention period: 1 March to 31 July 1997 | 1 ED; Pre-intervention (n=142), post-intervention (n=198) | Self-harm model of care | **System Performance:** referral rate, number of assessments,  **Patient Outcomes:** re-presentation rate | **System Performance:** referral rate increased, number of assessments increased,  **Patient Outcomes:** reduction in re-presentation rate |
| Opmeer, 2017, UK [59] | Pre-post (Quasi-experimental) | To increase the proportion of patients attending the ED after self-harm who receive a psychosocial assessment and reduce admissions to acute hospital beds to await Liaison Psychiatry Service assessment | Pre-intervention period: 1 January – 31 March 2014; post-intervention period: 1 January – 31 March 2015 | 1 ED; Pre-intervention (n=298), post-intervention (n=318) | Liaison Psychiatry Service | **System Performance:** the proportion receiving a psychosocial assessment, wait time for psychosocial assessment, wait time between medical and psychosocial assessment, left without being seen (LWBS), cost per attendance and cost per patient | **System Performance:** 11% increase in the proportion receiving a psychosocial assessment, reduced wait time for psychosocial assessment, decreased wait time between medical and psychosocial assessment, decreased cost per attendance and cost per patient  **Patient Outcome:** decreased LWBS |
| Xanthopoulou, 2022, UK [58] | Qualitative | To explore patient experiences of psychosocial assessment after presenting with self-harm/suicidality | October 9, 2018 -September 4, 2019 | 1 ED; Patients (n=28) | Liaison Psychiatry Service | **Patient Experience:** patient experience | **Patient Experience:** therapeutic conversations reduce distress and instill hope, formulaic assessments that focus on risks were tedious and generic |
| **Mental health presentation strategies** | | | | | | | |
| Adams, 2021, USA [62] | Time series (Quasi-experimental) | (1) improving treatment providers’ communications across the continuum of care, (2) enhancing communication between the mental health emergency department nurses and the on-call psychiatrists, (3) developing on-line decisional support to enhance communication, and (4) providing providers with feedback on the impact of changes | July 2009 – June 2011 | 1 ED; Not reported | 1. Decision support tools  3. Discharge and transfer of care | **System Performance:** mean revisit rate to psychiatric ED within 30 days of ED visit discharge in 2010 and 2011 | **System Performance:** mean revisit rate decreased |
| Alexander, 2020, Ireland [63] | Pre-post (Quasi-experimental) | To execute and describe a quality improvement process and to evaluate and quantify outcomes of the quality improvement process. | Not reported | 1 ED; 190 pre-mapping patients and 190 post-mapping patients were compared, 11 ED staff interviewees | 1. Discharge and transfer of care  2. Liaison Psychiatry Service  4. Decision support tools  5. Role changes and rostering | **System Performance:** ED LoS hours (IQR); Number of patients with ED LoS >24 hours %; Number of target goals met | **System Performance:** reduced ED LoS, five of six target goals were achieved with the ‘define medical screening’ goal removed after concerns were raised that could lead to rigid clinical approaches to screening. |
| Bistre, 2022, Israel [67] | Quasi-experimental | This study aims to compare the reliability and acceptability of psychiatric interviews using telepsychiatry (TP) and face-to-face (F2F) modalities in the emergency room setting | April – June 2020 | 1 ED; 38 patients; 20 received telepsychiatry then F2F, 18 received F2F then telepsychiatry | Telepsychiatry | **System Performance:** agreement between F2F and TP on disposition, diagnosis, interview duration, perceived certainty of decisions  **Patient Experience:** patient preference  **Staff Experience:** staff satisfaction | **System Performance:** no difference between the modalities for disposition. Partial agreement on diagnosis. Interview duration was shorter for the second half of participants compared with the first half  **Patient Experience:** patients preferred TP if it reduces wait time  **Staff Experience:** no difference between the modalities for staff satisfaction |
| Brainch, 2018, USA [73] | Pre-post (Quasi-experimental) | To examine the impact of this schedule change (10 h shifts, moved down to later in the day to address the higher patient volume) on residents and Psychiatric Emergency Service patients | January 2016 – February 2017 | 1 ED; 1847 patients; 32 residents | Role changes and rostering | **System Performance:** resident sense of well-being and burnout, wait time during high traffic hours, wait time for all hours, LoS during high traffic hours, LoS for all hours | **System Performance:** shorter duty-hours is not a singular factor for overall resident satisfaction. Other factors such as, timing of child daycare, being a morning person, and being able to experience natural daylight, play an important role, shorter wait time and length of stay |
| Braitberg, 2018, Australia [75] | Pre-post (Quasi-experimental) | To assess the impact of the introduction of the behavioural assessment unit (BAU) on patient outcomes, process and quality of care | 2015-2016 | 1 ED; 5428 patients | Specialised unit within the ED | **System Performance:** ED LoS, ED Waiting time, time to see Emergency Mental Health (EMH) Clinician, Number of code greys, number of restrictive (physical, mechanical, therapeutic) restraint | **System Performance:** reduced LoS, fewer code greys and restrictive restraints |
| Broadbent, 2002, Australia [64] | Pre-post (Quasi-experimental) | The implementation and assessment of impact on practice of a Mental Health Triage Scale (MHTS) in the emergency department. | November 2000 – April 2001 | 1 ED; 414 patients, 23 nurses | Decision support tools | **Staff Experience:** staff confidence, perception of the appropriateness of timeframe; acuity consistency between the two triage scales | **Staff Experience:** staff confidence increased, MHTS recognised patient acuity |
| Clarke, 2006, Canada [81] | Quasi-experimental | To enhance recognition of urgent mental health situations, reduce wait times at critical points in the patient journey through ED, reduce LWBS, reduce aggressive incidents, increase staff comfort with mental health presentations | 1 month | 1 ED | Education and Training | **System Performance:** mean LoS (hours), LoS in ED Admitted, LoS in ED discharged, LoS in ED LWBS, **Staff Experience:** triage nurse confidence | **System Performance:** reduced mean LoS,  **Staff experience:** increased nurse confidence |
| Eppling, 2008, USA [70] | Pre-Post (Quasi-experimental) | To reduce the number of inappropriate admissions, increase the number of in-network patients, and provide appropriate cost-effective care to the mentally ill. | May 2004- 2005 (month not reported) | 1 ED, number of patients not reported | Role changes and rostering | **System Performance:** number of admissions; security hours  **Staff Experience:** staff satisfaction | **System Performance:** 5% decrease in 24-hour or less admissions; reduction in number and hours of security staff on standby  **Staff Experience:** the program exceeds expectations |
| Gabet, 2020, Canada [82] | Cross-sectional | To understand the impact of a brief intervention team, crisis center team and a family-peer support team on MH services use and user needs | Innovations implemented in 2016, data collected January - December 2019 | 1 ED; patient and family: brief intervention team (n=44), crisis center team (n= 37), family-peer support team (n= 20), total = 101 | Intervention teams | **Patient Experience:** patient and family experience, service use, adequacy of care | **Patient Experience: s**taff were compassionate and sensitive to them, that they listened carefully and genuinely helped. Patients also reported receiving rapid treatment; the treatment steps were explained, subsequent appointments set, and information on MH services made available |
| Johnsen, 2007, Norway [84] | Pre-post (Quasi-experimental) | To increase patient knowledge, satisfaction, and treatment outcomes and decrease feelings of coercion | July 2000 – December 2002: Baseline 62 days, project period 378 days, follow-up period 60 days | 1 ED; Baseline (n=81), project period (n=577), follow-up (n=105) | Standardised protocol | **Patient Experience:** patient satisfaction, information quality, knowledge, coercion experience, | **Patient Experience:** no change in patient satisfaction, improved quality of information, increased patient knowledge, coercion decreased during study period then increased during follow-up period |
| Lauer, 2008, USA [80] | Pre-post (Quasi-experimental) | To enhance safety (reduce use of restraints, reduce elopements), decrease committal rates and increase referrals to appropriate levels of care | 2004-2005 | 1 ED; 2,099 patients evaluated for safety (restraints). 1,350 patients committed. number of patients referred to care not specified | Specialised unit within the ED | **System Performance:** use of restraints, referrals to appropriate care  **Patient Outcomes:** involuntary commitment | **System Performance:** reduced use of restraints, increase in referrals to appropriate care  **Patient Outcomes:** reduction in involuntary commitment |
| Maeng, 2020, USA [69] | Cohort | To determine if integrating Psychiatric Assessment Officers (PAO) and telepsychiatry reduces the number of ED revisits by patients presenting with behavioral health conditions | January 1, 2017 -December 31, 2018 | 3 EDs; intervention (n=452), control (n=761), total 1,213 patients | 1.Role changes and rostering  2.Telepsychaiatry | **Patient Outcomes:** ED re-visit rate | **Patient Outcomes:** reduced ED re-visit rate at 90 days and 180 days following indexed visit |
| McCurdy, 2015, USA [86] | Pre-post (Quasi-experimental) | To examine the rates of occurrence of seclusion and restraints before and after modifying unit design by the installation of a full-length glass, lockable door with a system that made it close automatically. | Baseline period: November 2011–December 2011; the second period (use of the Brøset) January 2012 – February 2012; third period (implementation of the door) April 2012–May 2012 | 1 ED; Baseline (n= 1,377); second period (n=1,389); third period (n=1,513), total 4,279 patients | Environmental design | **System Performance:** rates of seclusion and restraint | **System Performance:** reduced rates of seclusion and restraint |
| Mitchell, 2020, Australia [76] | Pre-post (Quasi-experimental) | To determine the impact of Psychiatric Assessment and Planning Units (PAPUs) on timely short-term inpatient care (less than 72 hours), reduce the demands on ED by actively seeking patients from the ED (primary vs secondary not specified). | Baseline: 1 September 2014 - 31 August 2015; Intervention: 1 September 2017  - 31 August 2018 | 3 EDs; PAPU patients (n=30), PAPU staff (n=30) were interviewed; 1,723 admissions to PAPU across the 3 sites | Specialised unit within the ED | **System Performance:** PAPU LoS  **Patient Outcomes:** readmission rate, adverse event  **Patient Experience:** patient experience  **Staff Experience:** staff experience | **System Performance:** average PAPU LoS breached the 4-hour target  **Patient Outcomes:** readmission rate varied, no adverse event  **Patient Experience:** PAPU perceived as a sanctuary with caring and receptive staff  **Staff Experience:** felt assisted by PAPU, didn’t resolve flow issues |
| Okafor, 2016, USA [79] | Pre-post (Quasi-experimental) | To increase access to appropriate mental health care for patients with behavioral health disorders seeking emergency care, and reduce waiting times for all patients in the ED | Pre-intervention: January 2011 – August 2011, Intervention: September 2011 to May 2012 | 1ED; Pre-intervention (n=4329), intervention (n=4867) | Specialised unit within the ED | **System Performance:** ED LoS, time to triage, disposition to discharge, admission to departure, Psychiatry LoS, LoS in restraints | **System Performance:** 4% reduction in ED LoS time, 67% decline in time to triage, 9% reduction in disposition to discharge time, 8% reduction in admission to departure time, 14% reduction in LoS and use of restraints |
| Poremski, 2016, Canada [87] | Qualitative | To facilitate the connection of frequent ED users with mental health or addiction challenges to appropriate community-based services | Interviews were conducted 6 months after baseline interview between August 2013 and December 2013 | 5 EDs; frequent ED users (n=83); service users interviewed (n=20), providers interviewed (n=13) | Case management | **Patient Experience:** working relationships, service navigation, support needs, barriers to engagement  **Staff Experience:** rapport, case management relationship, existing connections | **Patient Experience:** working relationships are important, service navigation is not easy, transition between service support is important, shame and stigma are barriers to engagement  **Staff Experience:** rapport is critical, case management is not a short-term relationship, service users have multiple existing connections that require significant coordination. |
| Reinfeld, 2023, USA [72] | Pre-post (Quasi-experimental) | To identify problematic areas of patient care, implement solutions, and examined their effectiveness | October 2020 – March 2021 | 1 ED; approximately 30 ED physicians | 1. Standardised protocol  2.Role changes and rostering | **System Performance:** time to consult, order to note completion, time to complete consults  **Staff Experience:** communication, perceptions of timeliness, attitudes towards providers, | **System Performance:** reduced time to consult, reduced order to note completion time, reduced time to complete consults  **Staff Experience: felt** communication improved, consults were seen in a timely manner, positive attitudes towards providers |
| Simpson, 2018, USA [65] | Pre-post (Quasi-experimental) | To demonstrate the feasibility and clinical impact of implementing standards for emergency psychiatric evaluations | Pre-implementation period: January – April 2017, Implementation period: May – June 2017, Post-implementation period: July – October 2017 | 1 ED; Pre-implementation (n=1896), post-implementation (n=1937) | 1. Decision support tools  2.Standardised protocol | **System Performance:** admission rates, 30-day revisit rate, median LoS | **System Performance:** no difference in admission rates, 30-day revisit rate, median LoS |
| Sinclair, 2006, UK [71] | Pre-post (Quasi-experimental) | To improve patient, staff, and organisational outcomes by making a psychiatric nurse available in A&E departments to assess and manage patients presenting with mental health problems | 1999, Pre-implementation period: 1-3 months; Implementation period: 4-6 months; Post-implementation period: 7-12 months | 2 EDs; Pre-implementation period: Survey (n=527), Hospital 1 (n=700), Hospital 2 (672); Post-implementation period: Survey (n=511), Hospital 1 (n=807), Hospital 2 (331); Total: Survey (n=1038), Hospital 1 (n=3021), Hospital 2 (1343) | Role changes and rostering | **System Performance:** number of patients assessed, accuracy of assessments, waiting times, onward referral  **Patient Outcomes:** revisit rates  **Patient Experience:** patient satisfaction  **Staff Experience:** staff views | **System Performance:** 90% of patients assessed, assessments were appropriate and good quality, no difference in waiting times, onward referral reduced LWBS rates  **Patient Outcomes:** no difference in revisit rates  **Patient Experience:** no difference in patient satisfaction  **Staff Experience:** personalities were main reason for success, though the psychiatric nurses felt isolated from the mental health team and out of touch with developments |
| Stover, 2015, USA [66] | Pre-post (Quasi-experimental) | To decrease psychiatric admission wait time from 10.5 to 8 hours and increase the proportion of patients discharged by 11 a.m. from 20% to 50% | Baseline data period: 6 months before March 2013, implementation phase: April 2013 – February 2014, post-implementation data collection period: 6 months before February 2014 | 1 ED; Pre-implementation period patients (n=304), post-implementation period patients (n=292) | Discharge and transfer of care | **System Performance:** average ED wait time for admission, discharge timeliness from the psychiatric unit, | **System Performance:** average ED wait time for admission decreased, proportion of patients discharged by 11 am improved |
| Vakkalanka, 2022, USA [68] | Descriptive | To explore the experience of implementing telepsychiatry services and to identify the utilization, barriers, and facilitators in these sites. | June 2019 - December 2020 | 5 EDs; patient encounters (n=4130) for patients (n=3932). Key informant interviewees (n=7) | Telepsychiatry | **Staff Experience:** facilitators, barriers, impacts | **Staff Experience:** increasing need, supporting infrastructure, straightforward processes, familiarity with technology, collaborative relationships, technical limitations, unclear processes, improved workforce and care delivery, satisfied patients and better access |
| Wand, 2001, Australia [74] | Cross-sectional | To evaluate the mental-health consultation-liaison nurse role in an inner-city teaching hospital in Sydney Australia. | 3 month pilot | 1 ED; Focus groups: nurses (n=22), survey: ED staff (n=53) | Role changes and rostering | **System Performance:** role utilisation  **Staff Experience:** difficulties working with patients, areas to be addressed, knowledge, confidence, skills | **System Performance:** well utilised and highly valued  **Staff Experience:** frustration at inability to help the patient during waiting times, limited vocabulary, need more training and clear protocols for incidents and guidelines, trouble communicating with non-compliant, intoxicated or paranoid patients, difficultly identifying appropriate follow-up, and risk of violence. The role provides a resource for all the above |
| Wand, 2021, Australia [83] | Realist evaluation | To describe and compare the case mix and outcomes of patients seen by the Mental Health Liaison Nurse (MHLN) team model of care and report on both patient and clinician acceptability of this ED-based service | Site A implemented MHLN in 2012. Sites B and C: Pre-implementation Period: December 2016 - March 2018. Data collection period: October 2018 - October 2019 | 3 EDs; Patients at site A (n=1,358), site B (n=1,339), site C (n=1,146), Total 3,843 patients | Intervention teams | **System Performance:** MHLN wait times, ED LoS, proportion referred to community services, incidents of near miss,  **Patient Experience:** patient acceptability of the model of care, LWBS  **Staff Experience:** clinician acceptability of the model of care | **System Performance:** Reduced wait times, reduced ED LoS, 43% referred to community services, one near miss incident at one site  **Patient Experience:** patient acceptability of the model of care, 0-4 LWBS  **Staff Experience:** clinician acceptability of the model of care |
| Woo, 2007, USA [77] | Quasi-experimental | To evaluate the benefits of the psychiatric emergency service (PES) model, in comparison to the model of the psychiatric consultant to the emergency department (the consultation model). | 2006 | 1 ED; retrospective chart review of 100 involuntary PES patients and 100 involuntary patients in the consultation model | Specialised unit within the ED | **System Performance:** timeliness of care, completion of assessment, pathology tests, use of seclusion  **Patient Outcomes:** follow-up care provided, readmission rate after 30 days  **Patient Experience:** elopement | **System Performance:** no difference in timeliness of care, greater completion of assessment, no difference in pathology tests, fewer use of seclusion or restraint in PES model  **Patient Outcomes:** no difference in follow-up care provided or readmission rate after 30 days  **Patient Experience:** les**s** elopement in the PES model |
| Zeitz, 2018, Australia [78] | Descriptive | To describe a suite of capacity management principles that have been applied in the mental health setting that resulted in a significant reduction in time spent in two emergency departments (ED) and improved throughput | Phase 1: July 2014 - May 2015; Phase 2: May 2015 - July 2015; Phase 3: July 2015 - December 2015 | 2 EDs; | 1.Standardised protocol  2.Case management  3.Specialised unit within the ED | **System Performance:** ED LoS | **System Performance:** reduced ED LoS |
| Zwank, 2020, USA [85] | Pre-post (Quasi-experimental) | To determine if eliminating the requirement for routine screening lab tests for patients admitted to the psychiatric service reduces lab test requests. | Pre-implementation period: December 15, 2017–April 15, 2018, post-implementation period: May 16–September 16, 2018 | 1 ED; Pre-implementation (n=886), post-implementation (n=1,024), Total 1,910 patients | Standardised protocol | **System Performance:** number of lab tests ordered, total charges for orders, ED LoS, proportion of patients with no blood lab orders, consultations, transfers,  **Patient Outcomes:** mortality | **System Performance:** decrease in the number of lab tests ordered, reduction in total charges for orders, mean ED LoS decreased, proportion of patients with no blood lab orders increased, no increases in consultations or transfers,  **Patient Outcomes:** no patient deaths |

Note. BAU, behavioral assessment unit; COWS, Clinical Opioid Withdrawal Scale; CRP, crisis response plan; ECG, electrocardiogram; ED, emergency department; EMH, emergency mental health; F2F, face-to-face; LoS, length of stay; LWBS, left without being seen; MH, mental health; MHLN, Mental Health Liaison Nurse; MHTS, Mental Health Triage Scale; MOUD, medications for opioid use disorder; O-HQIP, Opioid Hospital Quality Improvement Program OUD, opioid use disorder; PAO, Psychiatric Assessment Officers; PAPUs, Psychiatric Assessment and Planning Units; PES, psychiatric emergency service; PTSD, post-traumatic stress disorder SBIRT, screening, brief interventions and referral to treatment; SSI, Scale for Suicide Ideation; TP, telepsychiatry; VMAS, Visual Mood Analog Scale; WHO, World Health Organisation
